# The correlation between serum calcium levels and prognosis in patients with severe acute osteomyelitis

**DOI:** 10.3389/fimmu.2024.1378730

**Published:** 2024-06-06

**Authors:** Yunlong Liu, Yan Zheng, Sheng Ding

**Affiliations:** ^1^ Department of Pediatric Surgery, Women and Children’s Hospital Affiliated to Ningbo University, Ningbo, Zhejiang, China; ^2^ Department of School of Foundation, Zhejiang Pharmaceutical University, Ningbo, China

**Keywords:** serum calcium ion, acute osteomyelitis, critically ill, death, prognosis

## Abstract

**Objective:**

To explore the relationship between serum calcium levels and the prognosis of severe acute osteomyelitis, and to assess the effectiveness of calcium levels in prognostic evaluation.

**Methods:**

Relevant patient records of individuals diagnosed with severe acute osteomyelitis were obtained for this retrospective study from the Medical Information Mart for Intensive Care (MIMIC-IV). The study aimed to assess the impact of different indicators on prognosis by utilizing COX regression analysis. To enhance prognostic prediction for critically ill patients, a nomogram was developed. The discriminatory capacity of the nomogram was evaluated using the Area Under the Curve (AUC) of the Receiver Operating Characteristic (ROC) curve, in addition to the calibration curve.

**Result:**

The study analyzed a total of 1,133 cases of severe acute osteomyelitis, divided into the survivor group (1,025 cases) and the non-survivor group (108 cases). Significant differences were observed between the two groups in terms of age, hypertension, sepsis, renal injury, and various laboratory indicators, including WBC, PLT, Ca2+, CRP, hemoglobin, albumin, and creatinine (P<0.05). However, no significant differences were found in race, gender, marital status, detection of wound microbiota, blood sugar, lactate, and ALP levels. A multivariate COX proportional hazards model was constructed using age, hypertension, sepsis, Ca2+, creatinine, albumin, and hemoglobin as variables. The results revealed that hypertension and sepsis had a significant impact on survival time (HR=0.514, 95% CI 0.339–0.779, P=0.002; HR=1.696, 95% CI 1.056–2.723, P=0.029). Age, hemoglobin, Ca2+, albumin, and creatinine also showed significant effects on survival time (P<0.05). However, no statistically significant impact on survival time was observed for the other variables (P>0.05). To predict the survival time, a nomogram was developed using the aforementioned indicators and achieved an AUC of 0.841. The accuracy of the nomogram was further confirmed by the ROC curve and calibration curve.

**Conclusion:**

According to the findings, this study establishes that a reduction in serum calcium levels serves as a distinct and standalone predictor of mortality among individuals diagnosed with severe acute osteomyelitis during their stay in the Intensive Care Unit (ICU) within a span of two years.

## 1 Introduction

Acute osteomyelitis is a form of inflammation that occurs rapidly as a result of microbial infection, which leads to the destruction of bone tissue (1, 2). This infection can be localized to specific bone tissue or involve the bone marrow, bone tissue, periosteum, and adjacent soft tissues simultaneously (1). Inflammatory mediators and electrolyte imbalances further contribute to the progression of the underlying condition. As the infection progresses, osteomyelitis worsens, leading to persistent necrosis in the bones and tissues. This triggers a progressively escalating systemic inflammatory response, eventually resulting in multi-organ dysfunction, thus culminating in severe acute osteomyelitis (2). Patients with severe acute osteomyelitis face a high risk of developing sepsis, accompanied by multi-organ dysfunction, due to an uncontrolled inflammatory response (2, 3). Currently, the severity and prognosis of acute osteomyelitis infection are determined by assessing serum inflammatory markers such as white blood cell count, C-reactive protein (CRP), erythrocyte sedimentation rate (ESR), and wound microbial cultures.

Severe acute osteomyelitis patients often experience exacerbated systemic infection accompanied by multi-organ dysfunction, necessitating the assessment of disease severity and prognosis using additional indicators. The field of medical diagnosis for this condition is witnessing a growing trend in the utilization of various biological indicators, such as serum procalcitonin (PCT), serum-free calcium ions, N-terminal pro-brain natriuretic peptide (NT-proBNP), and plasma D-dimer (D-D). The selection of appropriate indicators for disease detection plays a paramount role in effectively managing critically ill patients with infections (4). Recent studies (3, 4) have highlighted the possibility of electrolyte imbalance in critically ill patients due to systemic inflammatory response. Among these imbalances, abnormal calcium metabolism is commonly observed, leading to the occurrence of hypocalcemia. It is widely acknowledged that the prevalence of hypocalcemia in critically ill infected patients is as high as 88% (4).

However, the precise mechanism responsible for hypocalcemia in individuals with severe acute osteomyelitis has not been fully elucidated. Limited scientific research has investigated the connection between serum calcium ion levels and the prognosis of mortality in patients with severe acute osteomyelitis. Therefore, further examination of the relationship between serum calcium ion concentration and the risk of mortality in individuals with severe acute osteomyelitis is of great importance in clinical practice. This study aims to evaluate the correlation between serum calcium ion levels and the risk of mortality in patients with severe acute osteomyelitis during the two-year period following admission to the intensive care unit (ICU).

## 2 Materials and methods

### 2.1 Data source

This retrospective observational study utilized medical records retrieved from the MIMIC-IV database. The study included a cohort of 1133 patients who were diagnosed with acute osteomyelitis. The clinical data of patients admitted to the ICU between the years 2008 and 2019 were extracted from the MIMIC-IV database. The authors received a certificate (No. 38821147) and access to the clinical database after completing the Collaborative Institutional Training Initiative online training course. It is important to note that this study strictly analyzed data from a publicly available anonymized database, MIMIC, and did not require ethical committee approval.

#### 2.1.1 Inclusion criteria

(1) We included patients who were diagnosed with acute osteomyelitis upon their initial admission to the intensive care unit (ICU) (2). Within 24 hours of their admission to the ICU, blood tests were performed (3). Participants who were over 18 years old were included in the study.

#### 2.1.2 Exclusion criteria

Patients with missing survival data and calcium ion assay results were excluded.

#### 2.1.3 Management of missing data

The MIMIC-IV database typically contains comprehensive demographic information. However, it is not uncommon to encounter missing data for laboratory tests and physiological items. Nonetheless, it would be highly inappropriate to exclude patients with incomplete data or variables as this would introduce substantial bias into the study. Given the absence of agreement on a universally accepted percentage for missing values, past research has employed thresholds of 60% and 70% to designate missing data (5, 6). However, in our investigation, we have opted for a threshold of 50%. Prior to fitting each model, we made the assumption that the missing data followed a Missing At Random (MAR) pattern (7, 8). The “norm. predict” method in Multivariate Imputation by Chained Equation (MICE) was utilized to handle missing values in the database. In this process, each variable was imputed based on the information from all other variables, effectively mitigating the influence of missing data. This approach ensures a thorough and dependable analysis of the dataset, enhancing its comprehensiveness and reliability.

### 2.2 Observation indicators (DATA)

This research study focused on analyzing various variables. The demographic characteristics of the population were taken into account, including gender, age, body mass index (BMI), marital status, race, admission and discharge times, and time of death. Additionally, the study examined complications such as sepsis, acute kidney injury (AKI), and hypertension. Laboratory parameters were also evaluated, including albumin, calcium, creatinine, glucose, hemoglobin, lactate, partial pressure of oxygen (PaO2), partial pressure of carbon dioxide (PCO2), red blood cell (RBC) and white blood cell (WBC) counts, C-reactive protein (CRP), alanine aminotransferase (ALT), aspartate aminotransferase (AST), alkaline phosphatase (ALP), platelet count, and wound microbiological detection. To categorize patients with acute severe osteomyelitis based on their survival status two years after discharge, they were divided into two groups: the survivor group and the non-survivor group. For patients with multiple hospital admissions, the data from their first admission was used. In cases where repeated examinations were conducted, the data from the initial examination within 24 hours after the first admission were utilized.

### 2.3 Statistical methods

The PostgreSQL database management system was utilized for data extraction and processing purposes. R software (Version 4.2.3) was employed to conduct statistical analysis. In order to assess the normality of the quantitative data, the Shapiro-Wilk test was employed. The results indicated a non-normal distribution in the quantitative data, which were represented as the median (quartiles) [M(Q1, Q_3_)]. Intergroup comparisons were performed using non-parametric tests such as the Mann-Whitney U test or Kruskal-Wallis test. Count data were presented as percentages (%25), and comparisons between groups were analyzed using the Pearson chi-squared test. Univariate and multivariate Cox regression models were employed to identify variables that were correlated with 2-year mortality in hospitalized patients. In accordance with the conventional variable selection process (9, 10), we initially performed univariate Cox regression analysis. Then, we proceeded to select variables that demonstrated statistically significant differences for inclusion in the multivariable Cox regression model. In addition, we evaluated the model’s adequacy by testing the proportional hazards assumption of the COX regression model.

We utilized the results derived from the multivariate Cox regression analysis to create a nomogram. Moreover, we constructed a receiver operating characteristic (ROC) curve and calculated the area under the curve (AUC) to assess the predictive performance of the nomogram. We validated the nomogram by employing the concordance index (C-index) and calibration curve. Any p-value below 0.05 was deemed as indicative of a statistically significant distinction.

## 3 Results

### 3.1 Baseline characteristics of the included patients

The study analyzed a total of 1,133 cases of severe acute osteomyelitis, with 1,025 cases in the survivor group and 108 cases in the non-survivor group. Of the cases examined, 756 were male and 377 were female. The average age of individuals in the survivor group was 59.0 years [with a range of 50.0 to 68.0 years], while in the non-survivor group, it was 74.5 years [with a range of 62.0 to 82.0 years].

We conducted stratified analysis on serum calcium ion, serum albumin, and serum hemoglobin. Patients with serum calcium levels below 9 mg/dl, albumin levels below 3.5 g/dl, and hemoglobin levels below 10 g/dl displayed a higher mortality rate within two years (P < 0.05). The overall two-year mortality rate among patients with severe acute osteomyelitis was 9.53% (108/1133). Significant differences were observed between the survivor and non-survivor groups in terms of age, hypertension, sepsis or septic shock, renal injury, and various laboratory indicators including white blood cell count (WBC), platelet count (PLT), serum calcium (Ca+), C-reactive protein (CRP), hemoglobin, albumin, aspartate aminotransferase (AST), alanine aminotransferase (ALT), and creatinine (P < 0.05). There were no statistically significant differences in race, gender, marital status, detection of wound microbiota, blood sugar levels, lactate levels, and alkaline phosphatase (ALP) levels. The baseline characteristics of the included patients are presented in **Table 1**.

**Table 1:** Demographic and clinical characteristics of the study population.

	Survival group	Death group	p
	N=1025	N=108	
Gender			0.601
female	344 (33.6%)	33 (30.6%)	
male	681 (66.4%)	75 (69.4%)	
Age (year)	59.0 [50.0;68.0]	74.5 [62.0;82.0]	<0.001
Lactate (mmol/L)	1.50 [1.20;2.10]	1.60 [1.20;2.12]	0.469
Hemoglobin (g/dL)	12.3 [10.9;13.5]	10.4 [9.28;12.4]	<0.001
Creatinine (mg/dL)	1.10 [0.80;1.50]	1.55 [1.00;2.73]	<0.001
Glucose (mg/dL)	135 [100;206]	126 [102;188]	0.573
WBC,109/L	8.80 [6.90;11.8]	9.60 [6.47;13.4]	0.037
Crp (mg/L)	56.0 [13.7;119]	77.9 [34.1;162]	0.001
ALT (IU/L)	21.0 [14.0;36.0]	18.0 [12.0;29.0]	0.032
AST (IU/L)	23.0 [17.0;35.0]	26.0 [19.0;40.0]	0.041
ALP (IU/L)	92.0 [70.4;123]	103 [71.5;143]	0.213
Albumin (g/dl)	3.60 [3.05;4.00]	3.20 [2.70;3.63]	<0.001
Ca (mg/dL)	8.90 [8.46;9.30]	8.60 [8.28;9.10]	0.001
Platelets,109/L	267 [206;345]	248 [189;308]	0.046
Aki			0.004
NO	788 (76.9%)	69 (63.9%)	
YES	237 (23.1%)	39 (36.1%)	
Hypertension			<0.001
NO	475 (46.3%)	70 (64.8%)	
YES	550 (53.7%)	38 (35.2%)	
Pathogenic bacteria			0.787
NO	36 (3.51%)	4 (3.70%)	
YES	989 (96.5%)	104 (96.3%)	
Marital status			0.165
NO	559 (54.5%)	67 (62.0%)	
YES	466 (45.5%)	41 (38.0%)	
Race			1.000
Other	315 (30.7%)	33 (30.6%)	
The white race	710 (69.3%)	75 (69.4%)	
Sepsis:			<0.001
NO	699 (68.2%)	53 (49.1%)	
YES	326 (31.8%)	55 (50.9%)	
Ca (mg/dL)			0.012
>11 mg/dL	41 (4.00%)	1 (0.93%)	
9~11mg/dL	444 (43.3%)	35 (32.4%)	
<9mg/dL	540 (52.7%)	72 (66.7%)	
Albumin (g/dL)			<0.001
>5.0 g/dL	5 (0.49%)	0 (0.00%)	
3.5~5.5g/dL	578 (56.4%)	36 (33.3%)	
<3.5g/dL	442 (43.1%)	72 (66.7%)	
Hemoglobin (g/dL)			<0.001
>15g/dL	81 (7.90%)	4 (3.70%)	
10~15g/dL	792 (77.3%)	67 (62.0%)	
<10g/dL	152 (14.8%)	37 (34.3%)	

WBC, white blood cell; AKI, acute kidney injury; AST aspartate aminotransferase; ALT, alanine aminotransferase. ALP, alkaline phosphatase; CRP, C-reactive protein.

### 3.2 Univariate and multivariate COX regression analysis

We performed both univariate and multivariate Cox regression analyses on baseline variables, laboratory indicators, and organ dysfunction to examine their impact on mortality within two years after admission. The primary endpoint of our observation was defined as mortality within the post-admission period. The findings from the univariate Cox regression analysis indicate that several factors were significantly associated with mortality in severe patients with acute osteomyelitis. These factors include age, hypertension, sepsis or septic shock, renal injury, white blood cell count, blood calcium levels, C-reactive protein (CRP), creatinine, albumin, and hemoglobin (p < 0.05). We shall now proceed to the multivariate analysis to further explore the independent effects of these variables on mortality in this patient population.

We constructed a multivariate Cox proportional hazards model to analyze the impact of various factors on survival time in patients with severe acute osteomyelitis. The variables included in the model were age, hypertension, sepsis, serum calcium ion levels, creatinine levels, albumin levels, and hemoglobin levels. The results showed that a history of hypertension had a significant effect on survival time, with a hazard ratio (HR) of 0.514 (95% CI 0.339–0.779, P = 0.002). Patients with sepsis had a significantly lower survival time compared to those without sepsis, with an HR of 1.696 (95% CI 1.056–2.723, P = 0.029). Furthermore, age, hemoglobin levels, serum calcium ion levels, albumin levels, and creatinine levels all demonstrated statistically significant impacts on survival time. The HRs for these variables were as follows: age (HR = 1.081, 95% CI 1.064–1.098, P < 0.001), hemoglobin levels (HR = 0.888, 95% CI 0.800–0.984, P = 0.024), serum calcium ion levels (HR = 0.731, 95% CI 0.564–0.946, P = 0.017), albumin levels (HR = 0.550, 95% CI 0.405–0.745, P < 0.001), and creatinine levels (HR = 1.192, 95% CI 1.099–1.293, P < 0.001). However, no statistically significant impact on survival time was observed for the other variables included in the analysis (P > 0.05). In summary, our multivariate Cox regression analysis revealed that age, hypertension, sepsis or septic shock, blood calcium levels, creatinine levels, albumin levels, and hemoglobin levels are all independent prognostic factors for severe acute osteomyelitis (P < 0.05), as summarized in **Table 2**.

**Table 2:** Cox regression analysis of risk factors for death within 2 years in ICU patients with severe osteomyelitis.

	Univariate analysis			Multivariate analysis		
	P	HR	95%CI	P	HR	95%CI
Age	<0.001	1.070	1.055~1.086	<0.001	1.081	1.064~1.098
Hemoglobin	<0.001	0.749	0.683~0.821	0.024	0.888	0.800~0.984
WBC	0.004	1.047	1.015~1.079	0.167	1.021	0.991~1.052
Ca	<0.001	0.646	0.510~0.818	0.017	0.731	0.564~0.946
Hypertension	<0.001	0.482	0.325~0.716	0.002	0.514	0.339~0.779
Albumin	<0.001	0.485	0.376~0.627	<0.001	0.550	0.405~0.745
Sepsis	<0.001	2.142	1.469~3.123	0.029	1.696	1.056~2.723
Aki	0.003	1.824	1.231~2.701	0.329	1.279	0.781~2.195
Creatinine	<0.001	1.163	1.094~1.237	<0.001	1.192	1.099~1.293
Crp	0.003	1.003	1.001~1.006	0.653	1.001	0.998~1.003
ALT	0.473	1.000	0.999~1.001			
AST	0.260	1.000	0.999~1.000			
ALP	0.291	1.001	0.999~1.002			
Platelets	0.415	0.999	0.998~1.001			
Lactate	0.358	1.094	0.904~1.324			
Pathogenic bacteria	0.903	0.940	0.346~2.550			
Marital status	0.129	0.740	0.502~1.092			
Race	0.951	1.013	0.673~1.525			
Glucose	0.454	0.999	0.997~1.001			
Gender	0.554	1.132	0.752~1.704			

### 3.3 Establishment of a nomogram model for acute osteomyelitis

**Figure 1:**
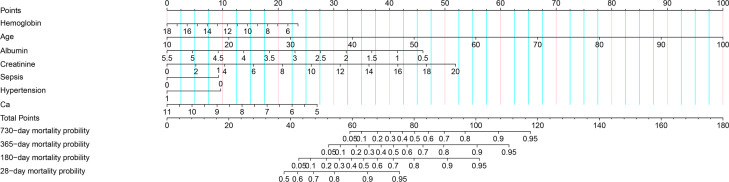
A nomogram of 2-year mortality risk.

Using the results of a multivariate COX regression analysis, we have developed a prognostic nomogram model that can accurately predict the 2-year mortality rate of critically ill patients with acute osteomyelitis. This nomogram is designed to establish scoring criteria for each individual prognostic parameter, assigning a score to represent its significance. The total score ranges from 0 to 180 points, as depicted in **Figure 1**. By utilizing this nomogram, we can now accurately assess the mortality risk based on the patient’s parameters upon admission.

### 3.4 ROC curve

**Figure 2:**
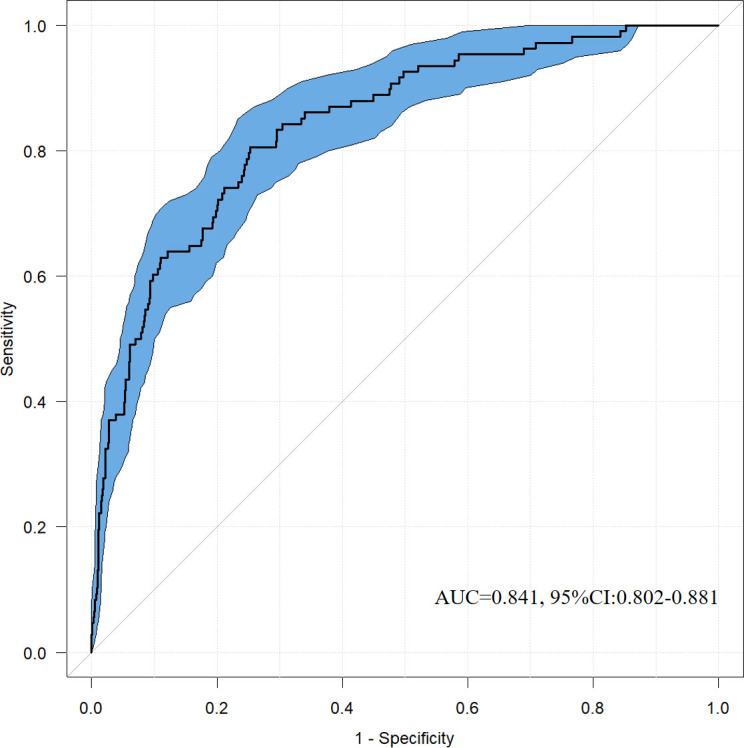
ROC curve evaluating the recognition ability of the nomogram.

The pROC package in R software was utilized to calculate the AUC. The AUC of the nomogram for prognosticating critically ill patients with acute osteomyelitis was found to be 0.841, with a 95% confidence interval (CI) from 0.802 to 0.881. This finding implies that the model predicts the likelihood of death in critically ill patients with acute osteomyelitis to be 84.1%, while exhibiting a specificity of 74.7% and a sensitivity of 80.6%. These outcomes substantiate the high accuracy of the nomogram (**Figure 2**).

### 3.5 Calibration curve

**Figure 3:**
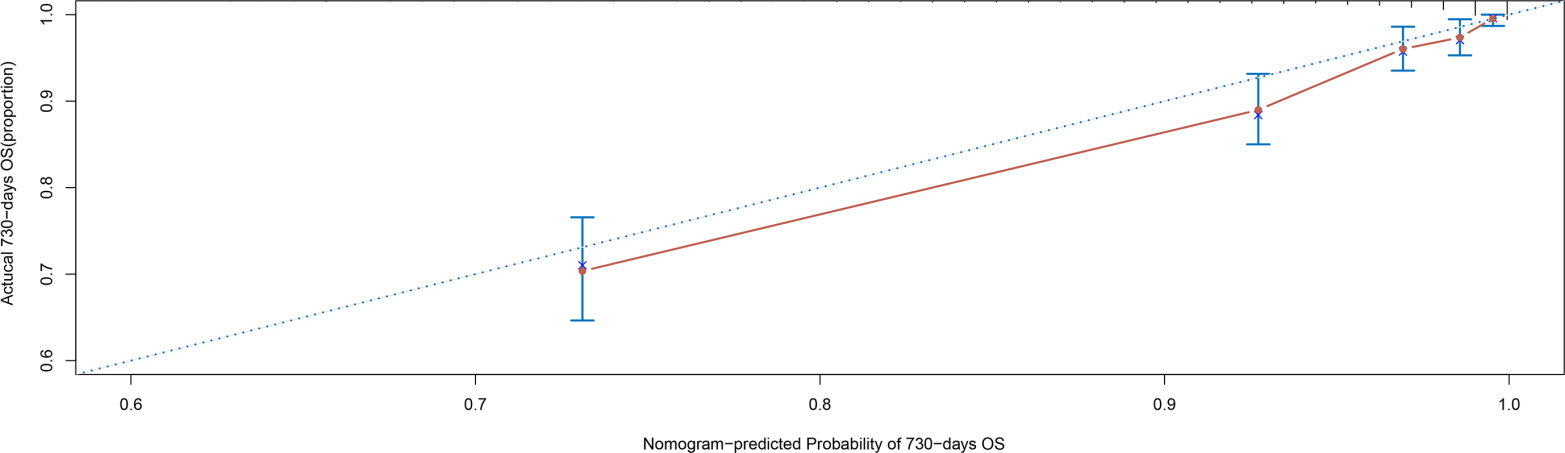
Calibration curve verified by the nomogram.

The accuracy of the nomogram model was assessed by examining its calibration curve. The findings revealed a close resemblance between the calibration curve and the ideal standard curve, indicating that the nomogram utilized in this study effectively predicted the 2-year mortality rate of critically ill patients diagnosed with acute osteomyelitis (**Figure 3**).

## 4 Discussions

Acute osteomyelitis, an inflammatory disease that affects the bone marrow and bone tissue, is caused by a bacterial infection (1). The prognosis of this disease is determined by several pathophysiological factors, including the infecting strains, the location and extent of the infection, the immune status of the host, the interventions used in treatment, and the occurrence of complications. Critically ill patients are particularly vulnerable to developing sepsis and multiple organ dysfunction as a result of uncontrolled inflammation (2, 3). Previous studies have demonstrated that the invasion of pathogens in patients with osteomyelitis triggers the activation of the monocyte-macrophage system, leading to secondary sepsis (11, 12). This activation leads to a significant release of inflammatory factors. Consequently, the levels of inflammatory factors in the bloodstream, such as PCT, CRP, NT-proBNP, as well as serum calcium, potassium, and other ions, become elevated in the body. It is crucial to promptly and effectively manage these influential factors in order to achieve a favorable prognosis.

Serum calcium levels are frequently employed as a diagnostic tool in clinical practice. Emerging research has revealed a link between numerous infectious diseases and a reduction in serum calcium levels (13–16). Evidence suggests that hypocalcemia is frequently observed in individuals with bacterial infections, highlighting the potential importance of calcium in the context of bacterial-associated infections (16). Additionally, abnormal serum calcium levels can manifest in a range of infectious diseases, including dengue fever and hepatitis A virus infection (17, 18).

The study findings demonstrate that patients with serum calcium ion levels below 9 mg/dL have a significantly higher mortality rate within two years (p<0.05). Additionally, higher infection severity is associated with an increased likelihood of organ dysfunction, resulting in a notable reduction in survival time. Among different ethnic groups, Caucasians exhibit higher prevalence rates compared to other ethnicities. These findings align with the medical information stored in the database. The majority of patients enrolled in the study come from European and American nations, where treatment and care protocols are tailored to the specific physiological characteristics of European and American populations. The results of the multivariate Cox regression analysis indicate that serum calcium ions independently contribute to the risk of mortality within two years after ICU admission in critically ill patients diagnosed with osteomyelitis. To accurately assess the prognosis of critically ill patients, a nomogram involving serum calcium ions was developed using independent predictive factors. The resulting AUC demonstrates excellent discriminatory capacity. This finding is consistent with previous research that has reported decreased serum calcium levels in patients with severe COVID-19 infection (13) and neonatal infections (14).

The management of trauma patients could greatly benefit from the application of nomograms, considering its significant clinical importance and promising prospects. Nomograms allow for the integration of various clinical parameters, enabling personalized assessment of patients’ conditions and prognoses. Through research, powerful nomogram models have been established, which combine factors like previous recurrence rates, epiphysis involvement, preoperative serum albumin levels, axial length of infectious lesions, lesion excision methods, and muscle flaps. These models robustly predict the risk of recurrence in chronic osteomyelitis of long bones (19). Additionally, Zhu et al. utilized nomograms to evaluate the risk factors for blood transfusion in elderly patients undergoing hemiarthroplasty for femoral neck fractures (20). The ability of nomograms to provide decision support to clinicians is of great importance, as it helps them formulate treatment plans more accurately. This, in turn, enhances treatment outcomes and improves patient survival rates.

Recent research studies (3, 4) have indicated that patients who are critically ill may experience an electrolyte imbalance during the systemic inflammatory response. One common electrolyte imbalance that often occurs is abnormal calcium metabolism, leading to hypocalcemia. A high prevalence of hypocalcemia, reaching up to 88% (4), has been observed in critically ill patients with infections. Another study (21) has revealed that 16.4% of patients with post-traumatic osteomyelitis (PTOM) have asymptomatic hypocalcemia before undergoing surgery. However, the potential diagnostic value and underlying mechanism of serum calcium levels in PTOM are not yet fully understood. A retrospective study (15) discovered that preoperative serum calcium levels in patients with PTOM were significantly lower compared to both the nonunion group and the normal union group (control group). Statistical analysis revealed that the average calcium level in the PTOM group was the lowest, measuring at 2.28 mmol/L (p<0.001), when compared to the nonunion group and the fracture normal healing group (control group). Furthermore, the study indicated that asymptomatic hypocalcemia could be used as a predictive factor for a poor prognosis in patients with PTOM.

In the human body, calcium ions are of utmost importance for various vital physiological processes. These processes include nerve conduction, muscle contraction, and diverse secretion mechanisms (22–24). Low levels of calcium in the blood can have significant effects. One such effect is the potential increase in nerve and muscle excitability, leading to convulsions and spasms. Additionally, it also enhances the excitability and conductivity of the myocardium, while reducing the influx of calcium. As a result, this condition prolongs the phase of action potential plateau, extends the refractory period, and diminishes myocardial contractility (25–27). Local tissue damage can disrupt the balance of calcium ions inside and outside cells, triggering a series of signal transduction pathways (28, 29). Calcium ions can activate signaling molecules such as calmodulin, influencing the proliferation, differentiation, and cytokine production of T cells and B cells, thereby regulating the intensity and persistence of immune responses (30, 31). A Mendelian randomization study indicated an association between elevated CD8^+^T cell counts and increased susceptibility to osteomyelitis, suggesting a causal relationship between higher genetic susceptibility with increased risk of osteomyelitis in individuals with higher circulating immune cell counts (32). Furthermore, calcium ions play a crucial role in inflammatory responses. They can activate multiple inflammatory signaling pathways, including NF-κB, promoting the release of inflammatory mediators such as interleukins and tumor necrosis factor, thereby exacerbating or mitigating inflammatory reactions (33). Notably, calcium ions can also regulate the permeability of endothelial cells, influencing the migration of white blood cells to sites of inflammation. By modulating immune signal transduction and inflammatory responses post-injury, calcium ions impact the function of immune cells and the progression of inflammatory processes, thereby exerting a significant influence on the prognosis of diseases.

The precise underlying mechanism responsible for hypocalcemia in critically ill infected patients remains elusive. Prior investigations (25, 34, 35) have proposed various potential factors, encompassing immune response, disrupted transport of intracellular and extracellular calcium ions, coagulation dysfunction resulting in calcium ion depletion, aberrant secretion and resistance to parathyroid hormone (PTH), hypoproteinemia, multiple organ dysfunction, and gastrointestinal dysfunction.

This study has identified that the serum calcium ion concentration is an independent risk factor for mortality in patients with severe acute osteomyelitis. The hazard ratio (HR) for this association is 0.731, with a 95% confidence interval (CI) of 0.564 to 0.946, and a p-value of 0.017. Hypocalcemia is more likely to result in multi-organ dysfunction, and the likelihood of death rises with the increasing severity of inflammation. There are several potential factors that could contribute to these findings.

Studies have indicated that the immune response to infection is significantly influenced by serum calcium and its regulatory factors (36, 37). The immune system actively combats infections (1, 2) and triggers inflammatory reactions. This process involves the release of various inflammatory cytokines by immune cells, such as tumor necrosis factor-alpha (TNF-alpha) and interleukin-1 (IL-1). These cytokines can potentially impact the secretion and function of parathyroid hormone (PTH). PTH plays a crucial role in maintaining calcium balance, and its inhibition may result in inadequate calcium release from the bones, thereby decreasing the concentration of calcium in the bloodstream.

Treatment of severe acute osteomyelitis often requires a combination of drug therapy and careful fluid management. It is important to note that certain medications, including antibiotics and diuretics, may have an impact on calcium metabolism and absorption. Additionally, the frequent use of acidic substances like Lactated Ringer’s Solution can potentially result in acidosis, which can further disrupt calcium protein binding and ion concentration (38–40). Critically ill patients may experience multiple organ dysfunction, including liver and kidney dysfunction (2). Infection, toxin accumulation, and other factors can adversely affect the kidneys. Renal dysfunction results in increased excretion of calcium and reduced reabsorption of calcium by the kidneys, leading to a decrease in the concentration of calcium in the blood (41). A significant difference in kidney damage was observed between the two groups in this study, indicating a notable statistical association (P<0.05). The multivariate Cox regression analysis revealed a statistically significant correlation between sepsis and survival time (HR=1.696, 95% CI 1.056~2.723, P=0.029). Furthermore, the analysis demonstrated a significant impact of creatinine on survival time (HR=1.192, 95% CI 1.099~1.293, P<0.001).

Patients who are critically ill often face numerous challenges such as tissue and organ ischemia, hypoxia, and acid-base imbalance. These conditions arise from the secretion of toxins and invasive substances by pathogens. As a result, the cell membranes may be damaged, leading to increased permeability and disruption in the transport of ions both within and outside the cells. Additionally, critically ill patients often experience adenosine triphosphate (ATP) depletion, which impairs the effectiveness of ATP-dependent calcium pump activity. Consequently, the removal of intracellular calcium becomes less efficient, resulting in a gradual buildup of calcium within the cells (26, 27, 42). Moreover, endotoxins and invasive enzymes can activate the coagulation and fibrinolysis system, causing systemic microvascular thrombosis. This process leads to a substantial depletion of coagulation factors and calcium ions, which are vital for the coagulation process. Consequently, there is an excessive consumption of calcium ions, leading to a decrease in the levels of serum calcium ions (43, 44). In a recent study, a significant statistical difference in platelet count between two groups was observed (P < 0.05). In cases of severe infection, the activation of neutrophil cells and the release of inflammatory factors can impair the body’s response to parathyroid hormone. This can result in reduced sensitivity of bone and renal tubular epithelial cells to parathyroid hormone, thereby affecting the mobilization of calcium from bones and the reabsorption of calcium in the kidneys. Furthermore, patients with severe infection often suffer from hypomagnesemia, which suppresses the secretion of parathyroid hormone (45). The early secretion of glucagon can also trigger the release of calcitonin, which inhibits bone resorption and lowers blood calcium levels (26, 46).

Recent research (47, 48) has uncovered that hypoalbuminemia is a common occurrence among critically ill patients. Moreover, changes in total serum calcium levels often parallel fluctuations in albumin concentration. When the levels of protein, particularly albumin, vary significantly, it affects the blood calcium levels accordingly. Specifically, for every 10 g/L decrease in serum albumin concentration, the serum calcium level diminishes by 0.2 mmol/L. The statistical analysis of this study reveals a significant impact of albumin on survival time (HR=0.550, 95% CI 0.405–0.745, P < 0.001). Additionally, patients may experience reduced gastrointestinal motility or impaired absorption function (49). The severity of these symptoms can vary depending on the type and severity of the primary infectious diseases. Prolonged and severe gastrointestinal dysfunction can result in reduced intake, absorption, and synthesis of vitamin D and calcium (27).

A stratified analysis was performed in this study to examine the levels of serum calcium ions, serum albumin, and serum hemoglobin. The results showed a significantly higher mortality rate within two years (p < 0.05) among patients with serum calcium levels below 9 mg/dL, albumin levels below 3.5 g/dL, and hemoglobin levels below 10 g/dL. This suggests that individuals with these low levels are at a heightened risk of mortality within a two-year timeframe.

To sum up, hypocalcemia in critically ill patients with acute osteomyelitis can be attributed to multiple factors. These factors encompass the inflammatory response of the immune system, local bone degradation, drug treatment and fluid regulation, acidosis, multi-organ dysfunction, as well as vitamin D insufficiency. Such factors can lead to various manifestations, including muscle spasms, neurological complications, and arrhythmias. Consequently, healthcare providers should closely monitor the calcium levels of critically ill patients with acute osteomyelitis and adopt appropriate measures to restore normal physiological function and alleviate symptoms.

## 5 Conclusion

This study has several specific limitations that need to be addressed. Firstly, in assessing calcium balance, serum calcium ion plays a crucial role. However, it is important to consider this parameter in conjunction with other key clinical indicators. Regrettably, the MIMIC-IV database does not offer the necessary data for conducting such analysis, which is a significant drawback of this study. Additionally, there is a substantial amount of missing data for blood phosphorus, potassium, and chloride levels. As a result, it was not possible to analyze these indicators and draw any relevant conclusions. This further limits the scope and comprehensiveness of the study. In addition, it is worth mentioning that this study was conducted in a retrospective manner, which could potentially lead to information bias as data was extracted from the database. Moreover, there is a scarcity of information regarding medication usage, surgical treatment approaches, and other interventions.

To summarize, this study proposes a correlation between lower concentrations of serum calcium ions and an increased risk of mortality. The severity of a patient’s infection is known to be connected to the level of calcium ions. As the level of free calcium ions decreases, there is a higher likelihood of the patient developing a severe infection or sepsis. In our study, we have developed a nomogram to predict the prognosis of individuals with severe acute osteomyelitis. This nomogram provides valuable guidance for doctors in customizing personalized prevention and treatment plans for critically ill patients, ultimately leading to a reduction in the mortality rate during their hospitalization. However, it is important to acknowledge the limitations of this study, and additional large-scale, multicenter studies are required to further explore unknown prognostic factors and improve the efficacy of the nomogram.

## Data availability statement

The original contributions presented in the study are included in the article/**Supplementary Material**. Further inquiries can be directed to the corresponding author.

## Ethics statement

Ethical approval was not required for the study involving humans in accordance with the local legislation and institutional requirements. Written informed consent to participate in this study was not required from the participants or the participants’ legal guardians/next of kin in accordance with the national legislation and the institutional requirements.

## Author contributions

YL: Conceptualization, Data curation, Methodology, Supervision, Writing – original draft, Writing – review & editing. YZ: Conceptualization, Data curation, Methodology, Resources, Writing – original draft, Writing – review & editing. SD: Formal analysis, Funding acquisition, Investigation, Writing – review & editing.
